# An association between colonic adenoma and abdominal obesity: a cross-sectional study

**DOI:** 10.1186/1471-230X-9-4

**Published:** 2009-01-15

**Authors:** YoungJoo Kim, YunJin Kim, Sangyeoup Lee

**Affiliations:** 1 Department of Family Medicine, Pusan National University Hospital, 1-10 Ami-dong, Seo-Gu, Busan 602-739, South Korea

## Abstract

**Background:**

Colorectal adenoma is a precursor lesion of colorectal cancer and thus, it is an important target for preventing colorectal cancer. Only a few studies suggest an association between colorectal adenoma and obesity, but results show considerable heterogeneity. In this study, we investigated the association between colorectal adenoma and waist circumference.

**Methods:**

165 adenoma cases and 365 polyp-free controls with a normal colon were compared in this cross-sectional study. Subjects underwent screening colonoscopy by experienced endoscopists. Demographic data, including smoking habit, were obtained by interview and waist circumference and anthropometric measurements were examined. Dietary intakes were evaluated using a food frequency questionnaire, and abdominal obesity was evaluated by measuring waist circumference. Statistical analysis was performed using SPSS for 13.0.

**Results:**

Age, waist circumference, and BMI were significantly higher in cases than controls. And smokers and men were more prevalent among cases than controls.

Among the abdominal obese subjects, 45.6% had 1 or more adenoma, and 9.0% of these had advanced adenoma, whereas among subjects with a normal waist circumference, only 25.7% had 1 or more adenomas. The prevalence of adenoma was higher among abdominal obese group (P < 0.05). Logistic regression analysis showed that abdominal obesity was associated with an increased risk of colorectal adenoma (OR, 2.74; 95% CI, 1.66~4.51 in men, OR, 2.58; 95% CI, 1.08~6.12 in women). These associations persisted even after adjusting for BMI. While BMI was found to be weekly associated with the risk of adenoma among men at the highest BMI levels. However, BMI was not associated with the risk for adenoma after adjusting for waist circumference.

**Conclusion:**

Our data suggest that abdominal obesity is associated with an increased risk of colorectal adenoma.

## Background

Colorectal cancer is one of the most common cancers, and several risk factors are known such as high-fat, low-fiber diet [1,2], low physical activity [2,3] and a family history [4]. In addition, obesity is a well-known risk factor of colon cancer. Obesity is a known risk factor for metabolic disease such as insulin resistance and is associated with an increased incidence or mortality rate for a number of cancers [5-7]. Furthermore, an association between obesity and cancer is being increasingly recognized and several mechanisms have been proposed to explain this association between obesity and cancer [8]. In particular, abdominal obesity is correlated with an accumulation of visceral fat, which is associated with the secretion of adipokines that may have a detrimental metabolic effect and increase insulin levels [9]. Several studies have concluded that colorectal cancer is related to insulin and insulin-like growth factors (IGFs) [10,11]. Prolonged exposure of colonocytes to IGF has been suggested to affect the proliferation and homeostasis of the colonic cells [12].

Colorectal adenoma is a precancerous lesion and the detection and removal of adenomatous polyps provides an opportunity for colon cancer prevention [13].

Therefore, it is important that we understand the etiology of colorectal adenoma and identify the risk factors associated with colorectal adenoma. Several studies have proposed that obesity is a risk factor for colorectal adenoma. It is known that there is an association between colorectal cancer and abdominal obesity [3,14-16].

In addition, recent studies have suggested that waist circumference show a greater association with colorectal adenoma than body mass index (BMI) [17]. However, only a few studies have investigated the association between abdominal obesity and colorectal adenoma and thus, evidence is insufficient to determine the association [18-20]. In this study, we investigated the association between colorectal adenoma and waist circumference as a measure of abdominal obesity.

## Methods

### Subjects

1187 subjects who underwent annual health check-ups and screening colonoscopy at Pusan National University Hospital in Busan, Korea, between 2003 and 2007 were enrolled in this cross-sectional study. Colonoscopy was performed by experienced endoscopists. Information about smoking habit were recorded during interview using a structured form. Dietary intakes were assessed using a food frequency questionnaire (FFQ) by a skilled dietitian. FFQ was designed to assess habitual diet during the previous 7-days and the daily intakes of food groups, energy, and nutrients were computed using an analysis program. Alcohol intake in current drinkers was calculated from consumption frequencies and types of alcoholic beverages (soju, beer, wine, whisky). Anthropometric measurements were examined by a single trained nurse. BMI categorization was based on the WHO Asia-Pacific classification for obesity: "normal" (BMI < 23.0), "overweight" (23 ≤ BMI < 25), "obese" (BMI ≥ 25) [5].

Colonoscopy was considered complete if reach the cecum, and preparation of the colon was adequate. We excluded 56 subjects who had findings that were not confirmed histologically or those with an incomplete colonoscopy examination and 318 subjects who had incomplete data. In addition, 283 subjects with cancer, inflammatory bowel disease, non-specific colitis, hyperplastic polyp or with previous history of bowel surgery or colorectal polyp were excluded. Finally, 165 cases with histologically confirmed colorectal adenoma and 365 controls with no remarkable abnormal finding on colonoscopy were evaluated in this study. The study protocol was approved by the local ethics and research committee at Pusan National University Hospital.

We collected data on the sizes and numbers of adenomatous polyp. Size of adenoma was determined using a measuring probe with 1-mm grading, which was inserted through an endoscopy. In cases with multiple adenomas, largest polyp was measured.

Advanced adenoma was defined as a polyp size of ≥ 1 cm or as a polyp with villous features or high-grade dysplasia.

### Assessment of waist circumference

Abdominal obesity was defined as a waist circumference of ≥ 90 cm for Korean men and ≥ 85 cm for Korean women [21]. Waist circumference were measured at the top of the hip bone by a doctor.

### Statistical analysis

Statistical analysis was obtained by SPSS for 13.0 (SPSS, Chicago, IL, USA). Age-and sex- adjusted mean values of baseline variables were compared between cases and controls by use of analysis of covariance models. Mean age was compared by 2-sample *t *test and the Chi-squared test was used to compare the sex and smokers in cases and controls. Chi-squared analysis was used to determine the significances of differences in the prevalence of adenoma between subjects with abdominal obesity and subjects with normal waist.

Binary logistic regression analysis was used to determine whether abdominal obesity was an independent risk factor of colon adenoma after adjusting for age, smoking, alcohol intake, total calorie intake and fat intake. Smoking habit was classified into three categories of 0, 1–500, and ≥ 501 cigarette-years and alcohol intake into three categories of 0, 27<, and ≥ 27 ml of alcohol per day. Odds ratios were used to measure the risk of adenoma attributable to BMI categories. Subjects were categorized by BMI into three groups: the normal group, the overweight group, and the obese group. P-value of less than 0.05 was considered to be statistically significant.

## Results

165 (31.1%) cases with colorectal adenoma and 365(68.9%) controls with a normal colon were analyzed. Mean age of these subjects was 52.3 ± 9.0 years and mean BMI was 24.6 ± 13.2 kg/m^2^. The age- and sex- adjusted variables of cases and controls are shown in Table 1. Waist circumference and BMI were significantly higher in cases than controls, but daily total calorie intake was lower in cases than controls (P < 0.05).

**Table 1:** Baseline characteristics of patients with colorectal adenoma and controls

	Patients with Adenoma* (n = 165)	Controls* (n = 365)	*P*^†^
Age(y)	55.8 ± 7.3	52.0 ± 9.1	< 0.001
Waist circumference(cm)c	87.2 ± 7.7	82.1 ± 8.4	< 0.001
Body mass index(kg/m2)	24.6 ± 2.8	23.8 ± 2.8	0.03
Daily total calorie intake(kcal/day)	1901.0 ± 347.1	1925.8 ± 394.6	< 0.01
Daily Fat intake(g/day)	45.1 ± 13.3	46.1 ± 16.3	0.07
Alcohol intake(ml)	30.7 ± 43.5	21.0 ± 36.2	0.39
Sex(% male)	78.2	56.4	< 0.001
Current smokers (%)	31.1	19.8	0.01

*, Mean ± S.D. for quantitative variables; number of subjects (%) for categorical variables;

†, obtained from analysis of covariance models for continuous variables, from *t*-test for age and from χ^2 ^test for categorical variables.

Mean age was significantly higher in cases than in controls (P < 0.001), and the proportions of smokers and men were significantly larger among cases (P < 0.05). However, no differences were observed between cases and controls in daily fat intake and alcohol intake (P > 0.05, Table 1).

Among cases, mean adenoma size was 0.5 cm and the mean number of adenomas was two. Histologically, tubular adenomas were in 91.6%, tubulovillous adenoma in 7.2%, and villous adenoma in 1.2%.

We compared the prevalence of colon adenoma in subjects with abdominal obesity and with a normal waist. The prevalence of nonadvanced and advanced adenomas were significantly higher in subjects with abdominal obesity (P < 0.05, Table 2). 45.6% of abdominal obese subjects and 25.7% of subjects with a normal waist had one or more adenomas. Furthermore, 9.0% of abdominal obese subjects and 3.4% of subjects with a normal waist had advanced adenoma.

**Table 2:** The prevalence of adenoma according to abdominal obesity No (%)

	Abdominal obesity group	Normal waist group*
No Adenoma	79(54.5)	286(74.3)
Nonadvanced adenoma	53(36.6)	86(22.3)
Advanced adenoma	13(9.0)	13(3.4)

Abdominal obesity, a waist circumference ≥ 90 cm for men and ≥ 85 cm for women according to Korean cutoff value;

*, χ^2 ^test

But prevalence of colon adenoma was not significantly different according to BMI (P > 0.05).

Also, Numbers of polyps are not significantly different between abdominal obese subjects and subjects with normal waist (P > 0.05).

Logistic regression analysis, controlled for age, daily total calorie intake, daily total fat intake, alcohol intake and smoking showed that abdominal obesity was associated with a significantly increased risk of adenoma (OR, 2.74; 95% CI, 1.66~4.51 in men, OR, 2.58; 95% CI, 1.08~6.12 in women). Furthermore, this significant association between abdominal obesity and adenoma was unchanged after adjusting for BMI (OR, 2.77; 95% CI, 1.54~5.00 in men, OR, 2.65; 95% CI, 1.02~6.90 in women, Table 3).

**Table 3:** Odds ratio of colon adenoma according to abdominal obesity.

		waist circumference(kg/m^2^)	
		
		normal (n = 145)	abdominal obesity (n = 385)
Male	Model 1 (95% CI)*	1.00 (referent)	2.74 (1.66~4.51)
	Model 2 (95% CI)^‡^	1.00 (referent)	2.77 (1.54~5.00)
Female	Model 1 (95% CI)*	1.00 (referent)	2.58 (1.08~6.12)
	Model 2 (95% CI)^‡^	1.00 (referent)	2.65 (1.02~6.90)

* Model 1, adjusted for age, smoking, daily total calorie intake, daily fat intake and alcohol intake;

‡ Model 2, additionally adjusted for BMI;

Abdominal obesity, a waist circumference ≥ 90 cm for men and ≥ 85 cm for women according to Korean cutoff value;

P value, obtained by binary logistic regression analysis

We also compared cases and controls according to BMI-specific category (normal, overweight, obese) using logistic analysis. Among the men, obese subjects were found to have a significantly higher risk of adenoma than normal BMI subjects (OR, 1.76; 95% CI, 1.00~3.11, Table 4). But after adjusting for waist circumference, the association between BMI and adenoma was not statistically significant (P > 0.05).

Among women, BMI was unrelated to risk of adenoma (Table 4).

**Table 4:** Odds ratio of colon adenoma according to BMI.

		Body mass index(kg/m^2^)		
		
		normal (n = 184)	overweight (n = 151)	obese (n = 195)
Male	Model 1 (95% CI)*	1.0(referent)	1.13(0.61~2.08)	1.76(1.00~3.11)
	Model 2 (95% CI)^‡^	1.0(referent)	0.93(0.49~1.75)	0.97(0.50~1.91)
Female	Model 1 (95% CI)*	1.0(referent)	0.89(0.34~2.34)	1.40(0.57~3.40)
	Model 2 (95% CI)^‡^	1.0(referent)	0.76(0.28~2.06)	0.93(0.34~2.55)

* Model 1, adjusted for age, smoking, daily total calorie intake, daily fat intake and

alcohol intake;

‡ Model 2, additionally adjusted for waist circumference;

BMI categorized according to WHO Asia-pacific classification,

P value, obtained by binary logistic regression analysis

## Discussion

In this study, we observed that an abdominal obesity is associated with an increased prevalence and a significant risk of colorectal adenoma. The abdominal obese subjects were found to be more that twice as likely to have colorectal adenoma. In addition, the prevalence of nonadvanced and advanced adenoma was found to be greater in abdominal obese subjects than in controls.

Many studies have been conducted on the association between colon cancer and obesity. One large cohort study showed that an increased waist circumference and an increased waist-to-hip ratio were strong risk factors of colon cancer because abdominal obesity is strong independent risk factors of insulin resistance and hyperinsulinemia, which have been suggested to be associated with colon cancer [18]. However, studies have been conducted on central obesity as a risk factor of colon adenoma, have been limited and the association remain controversial. In a Japanese report, high waist-to-hip ratio was found to be associated with an increased risk of colon adenoma in the men[19].

In addition, Giovannucci et al [18] reported that waist circumference and waist-to-hip ratio were also, strong risk factors of large colon adenoma, and other studies have reported similar findings [17,22,23].

The role of adiposity in the development of neoplasia of the colon is complicated and not well understood. Several mechanism have been suggested to explain why central obesity is a risk factor of colon adenoma; visceral fat influences on insulin resistance via a portal effect of free fatty acids, resulting in hyperinsulinemia and increased free insulin-like growth factor (IGF-I) [24]. Furthermore, IGF-I has been reported to stimulate the proliferation of colonic epithelium [12], and high affinity IGF-I receptors were present in human colon cancer cell lines and in resected human colon cancers [25] and thus, suggesting the relation IGF-I with the colon neoplasm [26-28]. In addition, decreased adiponectin levels caused by central obesity may also be associated with colon adenoma, because adiponectin participates in the regulation of inflammation and apoptosis by inhibiting nuclear factor κB signaling [29].

On the other hand, in the present study, BMI was not significantly associated with colon adenoma after adjusting for waist circumference, which concurs with the findings of a previous studies. Otake et al suggested that visceral fat tissue rather than whole body adipose tissue is associated with colorectal adenoma [20] and Koichi et al demonstrated that BMI was not associated with adenoma risk independently of waist-to-hip ratio [17]. Also, the majority of studies have found no significant association between the BMI and colorectal adenoma in women [30-33]. The pattern of body fat distribution is one explanation for gender difference in the association between BMI and adenoma. This findings suggest that adipose tissue distribution may be the important factor in the association between obesity and colorectal adenoma. In addition, waist circumference is related to colorectal adenoma more strongly than BMI.

This study has several limitations. First it is intrinsically limited by its cross-sectional design. Second, data about physical activity levels and family history were not collected. Nevertheless, our findings demonstrate that abdominal obesity is associated with colorectal adenoma, and this is in agreement with the previous studies. This result suggest that central obesity is a better and more important indicator of the colon cancer and precancerous lesions than general obesity. This may help to guide strategies targeted toward prevention of colon cancer.

In conclusion, the present study shows that high waist circumference is associated with an increased risk of colorectal adenomas. And this finding suggest abdominal obesity is a risk factor of colorectal adenomas.

## Conclusion

In conclusion our data suggests that abdominal obesity in Korean tends to be associated with an increased risk of colorectal adenoma. In the future, further studies are needed to clarify the role of abdominal obesity in the development of colorectal adenoma.

## Competing interests

The authors declare that they have no competing interests.

## Authors' contributions

YJK was responsible for the design of the study, analyzed data and drafted the manuscript. YJK collected the data and SYL advised for modifications in the statistical analysis and manuscript. All authors read and approved the final manuscript.

## Pre-publication history

The pre-publication history for this paper can be accessed here:

http://www.biomedcentral.com/1471-230X/9/4/prepub
